# Advances in Spiropyrans/Spirooxazines and Applications Based on Fluorescence Resonance Energy Transfer (FRET) with Fluorescent Materials

**DOI:** 10.3390/molecules22122236

**Published:** 2017-12-18

**Authors:** Hongyan Xia, Kang Xie, Gang Zou

**Affiliations:** 1 Guangdong Provincial Key Laboratory of Micro-nano Manufacturing Technology and Equipment, Guangdong University of Technology, Guangzhou 510006, China; xiahy@mail.ustc.edu.cn; 2 CAS Key Laboratory of Soft Matter Chemistry, Department of Polymer Science and Engineering, iChEM, University of Science and Technology of China, Anhui 230026, China

**Keywords:** spiropyran, spirooxazine, fluorescence materials, structural transformation

## Abstract

Studies on the following were reviewed: (1) the structure of spiropyrans and spirooxazines (two kinds of spiro compounds) under external stimuli and (2) the construction and applications of composite systems based on fluorescence resonance energy transfer (FRET) with fluorescent materials. When treated with different stimuli (light, acids and bases, solvents, metal ions, temperature, redox potential, and so on), spiropyrans/spirooxazines undergo transformations between the ring-closed form (SP), the ring-opened merocyanine (MC) form, and the protonated ring-opened form (MCH). This is due to the breakage of the spiro C–O bond and the protonation of MC, along with a color change. Various novel, multifunctional materials based on photochromic spiropyrans and spirooxazines have been successfully developed because of the vastly differently physiochemical properties posssed by the SP, MC and MCH forms. Among the three different structural forms, the MC form has been studied most extensively. The MC form not only gives complexes with various inorganic particles, biological molecules, and organic chemicals but also acts as the energy acceptor (of energy from fluorescent molecules) during energy transfer processes that take place under proper conditions. Furthermore, spiropyran and spirooxazine compounds exhibit reversible physicochemical property changes under proper stimuli; this provides more advantages compared with other photochromic compounds. Additionally, the molecular structures of spiropyrans and spirooxazines can be easily modified and extended, so better compounds can be obtained to expand the scope of already known applications. Described in detail are: (1) the structural properties of spiropyrans and spirooxazines and related photochromic mechanisms; (2) composite systems based on spiropyrans and spirooxazines, and (3) fluorescent materials which have potential applications in sensing, probing, and a variety of optical elements.

## 1. Introduction to Spiropyrans and Spirooxazines

**Scheme 1:**
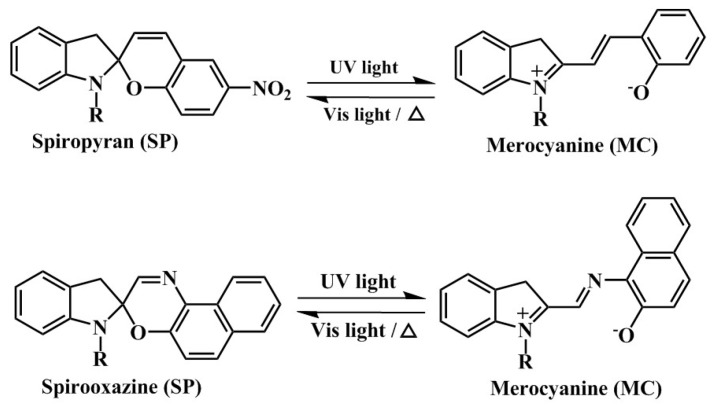
Reversible structural transformations of spiropyrans and spirooxazines.

Spiropyran and spirooxazine compounds can undergo reversible structural transformations under the influence external stimuli; this induces a color change, as well as changes in their physical and chemical properties [1,2]. Spiropyrans and spirooxazines are the most investigated photochromic spiro compounds [3,4]. It is reported that spiropyrans and spirooxazines could also respond to other external stimuli such as thermal effects, pH, stress, and so on [5,6]. The structural transformation processes of spiropyrans and spirooxazines are shown in Scheme 1 [7]. In order to be consistent, the terms “spiropyran’’ and “spirooxazine” refer to both the ring-closed and ring-opened form of isomers, where the ring-closed form is abbreviated as ‘‘SP’’ and the ring-opened form is abbreviated as ‘‘MC’’. After UV light irradiation, spiropyrans and spirooxazines transform from the ring-closed SP form to the ring-opened MC form. Here the MC form represents an equilibrium state comprised mainly of quinoidal and zwitterionic forms, depending on the polarity of the environment and the electronic properties of the substituents. For example, when spiropyrans and spirooxazines are in a polar environment and have electron-withdrawing substituents, the main equalibrium form is the quinoid one; however, a zwitterion is the main equalibrium form in a non-polar environment and when the substituents are electron-donating. Different equalized forms can undergo reversible conformational transformations when the environmental parameters change [8]. The structural transformation from SP (which is relatively apolar) to MC is accompanied by an increase of polarity [9,10,11]. When treated with visible light irradiation or heating, MC reversibly reverts back to SP.

As early as 1952, Hirshberg and Fischer had reported the photochemical reactions and photochromic phenomena of spiropyrans and spirooxazines; after UV light irradiation, these photochromic spiro compounds exhibit characteristic color changes (from colorless to purple) due to the cleavage of the spiro C–O bonds, producing a rearrangement of molecular conformations and electrons [12]. The purple form MC, which is obtained from the ring-opened reaction, has intense absorption bands in the visible light region. Different substituents and environments will lead to a blue shift or red shift of the absorption peaks. In order to gain more insight into the nature of the photoisomerization of spiropyrans and spirooxazines, Hirshberg et al. carried out in-depth studies (using different structures, environments, kinetics, and mechanisms, and so on) to analyze the above reaction [13,14,15]. Hirshberg suggested that this kind of spiro compound can be used as photochemical memory and in variable density optical shutters. This caused worldwide interest in industry and academia; plenty of research achievements were published in this area from 1948 to 1970. Bertelson summarized the study of spiropyrans in 1971 [16]. Due to the development of various characterization methods, the photochromic processes of spiropyrans and spirooxazines have been studied thoroughly. The progress of this has been extensively reviewed by Guglielmetti in 1990 [17].

In recent years, multifunctional materials constructed from spiropyrans and spirooxazines have been applied in many fields, for instance: chemical sensing [18,19,20,21], rewritable data storage [22,23], biological imaging [24], optical devices [25], and so on. This was because different spiropyran and spirooxazine isomers could interact with various materials: (1) inorganic particles such as metal ions, quantum dots (QDs), and up-conversion nanoparticles (UCNPs); (2) biological molecules such as DNA and proteins; and (3) organic systems such as organic small molecules and polymers. Klajn has comprehensively reviewed dynamic materials based on spiropyrans. In this review, the authors introduced the structural transformation processes and aggregation of the ring-opened isomers of spiropyrans in detail. Then, the dynamic materials based on spiropyrans and various surfaces (composed of polymers, nanoparticles, biomacromolecules, and so on) were summarized. This kind of molecular switch that occurs with spiropyrans and spirooxazines) endowed many composite systems with new properties that have potential applications in bioimaging, energy transfer, environmental probes, and more [26].

In addition, the zwitterionic MC form can act as the energy acceptor to quench the fluorescence of nearby energy-matched fluorophores (serving as energy donors); thus, treating the spiro compounds with external stimuli confers the whole system with switchable fluorescence properties. Raymo and colleagues completed a lot of work in this area. For example, in 2009, they reported the fluorescence switches based on spiro-compounds. Functional spiro components were incorporated into molecules or assemblies to modulate the fluorescence intensity or fluorescence wavelength of the whole system (intermolecular, solution, film, and nanostructured constructs). The modulation mechanism was discussed, including the conjugation, electron transfer and energy transfer. The efficiency of fluorescence modulation relied on the state of functional spiro components [27]. Yildiz and colleagues illustrated the design of many nanostructured spiropyran-based constructs through covalent or non-covalent connections, indicating that the above modulation principles can be widely used in species ranging from relatively small molecules and assemblies to nanostructured composite systems. The reversible structural transformations of spiropyrans were used to quench or restore the fluorescence of fluorophores. These studies brought about the precise control of optical signals, which could be used to fabricate novel smart materials [28]. In order to apply this to molecular devices based on the above modulation process, Guo et al. synthesized a new dyad by attaching two spiropyran units to one fluorescein unit. They constructed integrated logic gates with a string of three binary inputs and a specific output which allowed the processing and communicating of information at a single molecule level [29].

## 2. Structure and Mechanism of Photochromism

### 2.1. Structure of Spiropyrans

**Figure 1:**
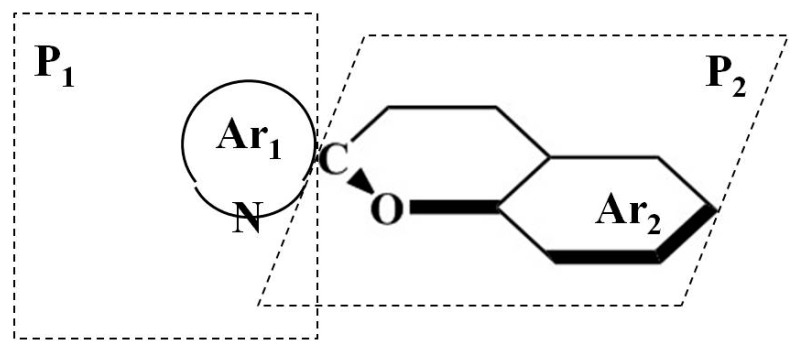
Basic structure of spiropyran.

Spiropyran is a general term for compounds which have two heteroaromatic rings (one is a pyran ring) and the two rings connected through a sp^3^ hybridized spiro C atom [18,30]. The basic structure is shown below (Figure 1).

Ar1 and Ar2 can represent benzene, naphthalene, anthracene, indolinol, thiophenol rings, or other aromatic rings (including heterocyclic rings). The most investigated spiropyran is the indolinospiropyran, in which Ar1 represents indolinol rings. Due to the sp^3^ hybridized spiro C atom connection, the two rings in the molecule are orthogonal to each other and there is no conjugation; this ring-closed form is usually called the SP form. There are electron transitions among the two rings of these ring-closed spiro molecules; thus, most of the ring-closed spiropyrans’ absorption peaks appear in the UV spectral region (generally within 200–400 nm) and they are colorless. After UV light irradiation, the configuration of the ring-closed spiro molecules and the distribution of electrons vary greatly due to the cleavage of the C–O bond. The original orthogonal two rings become coplanar and the whole molecule forms a large conjugate system. It is accompanied with a big red shift of the absorption spectra (appearing within the 500–600 nm range), resulting in a transformation from a colorless to a colored form. The C–O broken structure is called the ring-opened form or colored form, which is similar to the merocyanine dyes; thus, it is always called photomerocyanine (or MC). If treated with visible light or heating, the MC form returns to the SP form due to the ring-closing reaction, forming a typical photochromic system.

The spiropyran could maintain its photochromism properties whether it is in solution, a solid matrix (such as silica gel/resin/polymer), or a membrane. Other photochromic compounds rarely undergo structural transformations in the solid state because of a lack of free volume. However, Harada et al. reported the photochromic phenomena of several kinds of spiropyrans and spirooxazines in the solid state at low temperature. At 90 K, the spiropyran and spirooxazine crystals showed characteristic color changes (from colorless to colored) under UV light radiation. Decreasing the temperature could enhance the photochromic properties; the reason for this was attributed to the more stable photoinduced merocyanine which forms at low temperatures, resulting in the suppression of color fading [31].

**Scheme 2:**
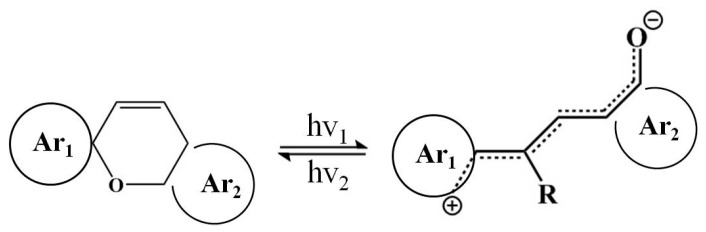
Switchable photoreaction formula of spiropyrans.

The reaction process can be expressed in the following scheme (Scheme 2). It should be pointed out that MC is comprised of a balanced mixture of ring-closed and ring-opened forms. For simplicity, the equation below only depicts one of these forms.

### 2.2. Photochromic Mechanisms of Spiropyrans

Flash photolysis was used to study the mechanism of photochromism for spiropyrans [32]. First, the ring-closed spiropyran forms the first excited singlet state under UV light radiation, then, the spiro C–O bond breaks, generating the ring-opened intermediate form. Finally, it decays into the various equilibrium states of the MC form [5,33]. The characteristic absorption of the intermediate form is observed in the 430–450 nm; its lifetime in solution ranges from 10^−8^ s to 10^−3^ s. It maintains the orthogonal orientation of the two rings [5,33]. Kholmanski studied the potential energy curve of the ground state and the excited state of spiropyrans, trying to explain the photochromic mechanisms [34]. It is reported that it was not only the singlet state but also the triplet state that was involved in the photoisomerized reaction of spiropyrans. During the photoisomerization reaction, the involvement of singlet state and triplet state essentially depends on the characteristics of the molecular skeleton and the position and properties of the substituents on the rings. As a reversible photochromic system, it must be controlled so that it cannot produce free radicals and ions or dipole intermediates during the photochemical reaction. Otherwise, it is difficult to control the thermal stability or side effects of the whole reaction, which reduces its practical value.

Absorption spectra of ring-closed spiropyrans are minimaly affected by environmental factors (including temperature, concentration, solvent, etc.), while the ring-opened forms are very sensitive. Generally, as the temperature decreases, the absorption intensity of the long-wave band for ring-opened spiropyrans reduces and the absorption intensity of the short-wave band accordingly increases. As the polarity of the solvent increases, absorption peaks in the visible light range show blue shifts, there is a decrease in the extinction coefficient, and the peaks simultaneously widen. According to the above behaviors, it is possible to determine that the dominant structure of the ring-opened forms is the zwitterion form.

### 2.3. Structure of Spirooxazines

**Figure 2:**
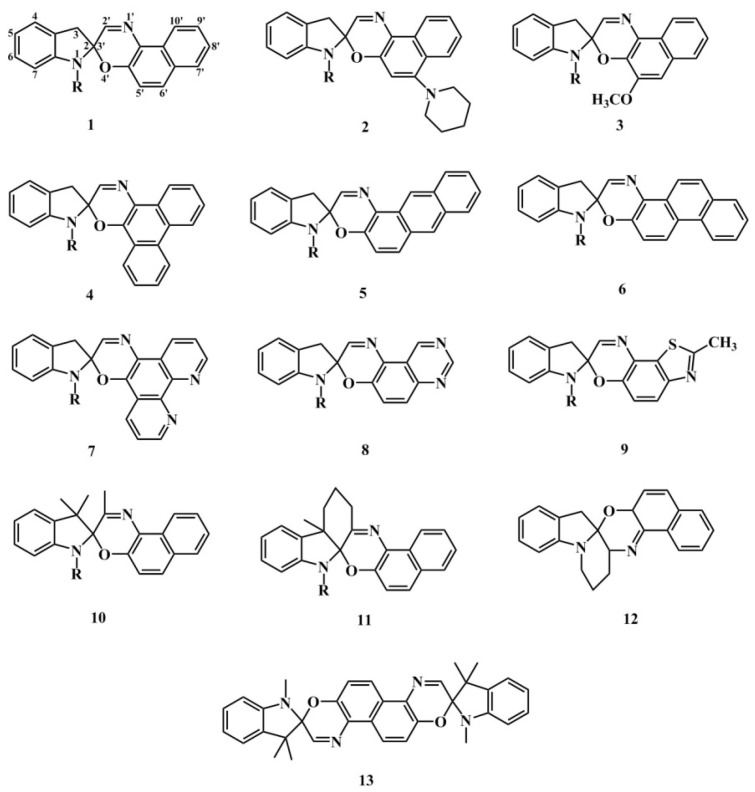
Different indolinospirooxazine structures.

The structure of spirooxazines is very similar to that of spiropyrans. Therefore, the synthesis methods, absorption properties, and the mechanism of photochromism are also very similar to those of spiropyrans. The photochromic phenomena of spirooxazines were first reported by Fox [35], who synthesized the first spirooxazine compound and observed that a toluene solution of this compound turned blue when irradiated under UV light. They also tried to prepare benzene substituted spirooxazines from nitrophenol but failed because these were unstable. Then, some spirooxazine derivatives were synthesized by Ono et al. [36]. Hovey et al. studied the influence of different substituents on the properties of spirooxazines [37]. To date, different spirooxazine structures have been successfully synthesized. Taking indolinospirooxazine as an example, there are several common types of structures as shown below (Figure 2).

The photochromic responses of these compounds are significantly improved by 9-methoxy and 8-bromo substituents, but these have little influence on the absorption spectra in the visible light range. Interestingly, the 6-amino substituent causes a blue shift of the absorption spectra of compound **2** (Figure 2) by 30–40 nm towards the visible light region. The 5-methoxy substituent will also affect the location of absorption peaks for compound **3**. Hovey et al. have also studied the influence of substituents in the indolino ring on the properties of these compounds.

Various spirooxazines had been studied in depth. The indolino ring and spirooxazine of compounds **11** and **12** are linked through bridged chains; thus, even if the breakage of the spiro C–O bond occurs, the whole configuration remains rigid. We can also observe the existence of the ring-opened forms. Fan et al. studied the photochromism of compounds **1**, **10**, **11** and **12** by laser flash photolysis technology [38]. For compound **10**, not only the normal chromophore can be measured, but a new unstable short life intermediate—named charge separated and distorted intermediate (CT)—is also obtained. Compound **11** is a new kind of fused spiro compound where only the CT can be obtained and which has a lifetime of about 120–150 s. Ring-opened reaction of compound **12** only involves the single excited state; the rigidity of the bridged ring hinders the rotation and recombination of other chemical bonds, resulting in a slow generation of the chromophore [39].

### 2.4. Photochromism of Spirooxazines

In-depth basic research on the photophysics and photochemical processes of spirooxazines has been carried out. Schneider et al. studied the mechanism of photochromism of spirooxazines by picosecond time-resolved absorption spectra, as well as time-resolved spontaneous and coherent anti-stokes Raman scattering [40]. Kellmann and colleagues studied the light physics and dynamics of spirooxazines in 1989 [41]. Pottier et al. studied the influence of substituents, heteroatoms, and solvents on the absorption spectra and photochromic kinetics of the ring-opened spirooxazines [42,43]. Wilkinson et al. studied the process of photochemical formation for ring-opened spirooxazines in different solvents through picosecond transient absorption and picosecond time-resolved resonance Raman in 1996 [44]. Malatesta pointed out that there existed a key intermediate during the photodegradation and that such an intermediate can be produced in photochromic processes, even without oxygen [45,46]. They also found the MC structure of ring-opened spirooxazines and a darker free radical product [47]. Malatesta et al. studied the photochromism of spirooxazines theoretically by means of Hartree-Fock SCF quantum chemistry calculations; the obtained results matched the experimental data well [46].

**Scheme 3:**
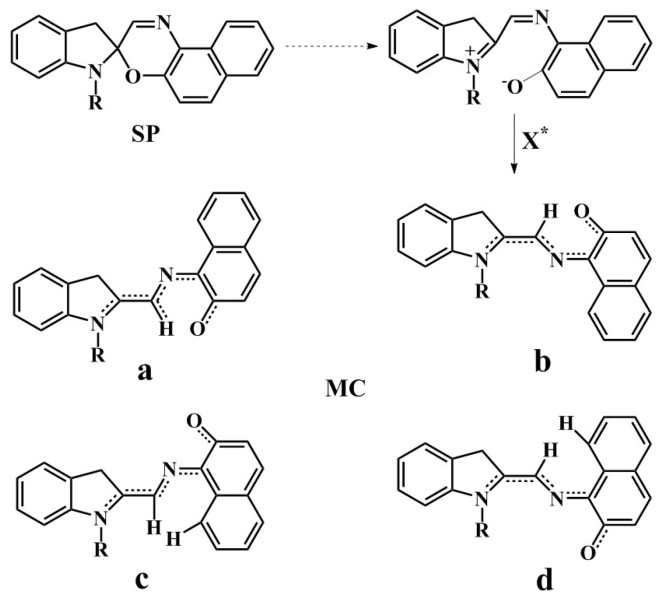
Structural transformation process of spirooxazine **1**.

Photochromic spirooxazine products are also called photomerocyanines (MC), because the mechanism is the same as that of spiropyran. MC is a the mixture of various isomers. It has been reported that there exist at least four stable isomers during the photochromic process. Taking compound **1** as an example, the reaction process is shown in Scheme 3.

Using the picosecond resolution spectroscopy technique, rupture of the spiro C–O bond happens in two ps after UV light irradiation. The initial light product is a non-planar intermediate X* (with a lifetime of about 3–11 ps) which then decays to MC [41]. The lifetime of X* is shortest in the polar aprotic solvents, a little longer in the polar protic solvent, and longest the nonpolar solvents. The whole color changes very quickly and, in about 20 ps, MC reaches the thermodynamical quasi-equilibrium state. The presence of oxygen has no effect on the whole transient process, indicating that the photochromic reaction of indolinoline spirooxazines proceeds in the singlet state. Contrary to the opened ring of spiropyran, the opened spirooxazine ring possesses a positive solvent color effect whereby, when the solvent polarity increases, there is a red shift of the absorption spectra. Thus, the main existing form of the ring-opened spirooxazines is the quinone structure caused by heteroatoms which are involved in electron delocalization.

The quantum chemistry calculations show that MC(a) and MC(b) in the Scheme 3 are the most stable planar isomers, with absorption peaks at 599 nm and 605 nm, respectively. The absorption peak of MC(c) is the shortest (at about 567 nm). MC(c) has the largest dipole moment, showing the maximum solvent effect. Since MC is a mixture of various isomers, the absorption band is relatively wide. Although the structure of spirooxaine is similar to that of spiropyran, there still exists some differences between these two kinds of spiro compounds. Tyer and Becker made a comparative study for the absorption spectra of spiropyrans and spirooxazines before and after photoisomerization. Because they posses the same benzopyrene and indoline ring, most of the absorption peaks of spiropyrans and spirooxazines were the same. However, for spirooxazines, there existed characteristic absorption peaks for the electron transitions of naphthoxazines. When the hydrogen atom on the indoline ring was substituted by alkyl, alkoxyl and halogen, the absorption spectra varied greatly; for example, the two absorption peaks in the long wavelength region of 5-alkoyl-substituted spirooxazines changed to a single peak [48].

Some spirooxazine compounds also have the property of thermochromism. For example, a colorless ethanol solution of compound **1** (with a concentration of 10^−6^ mol/L) turns blue when concentration is increased. The higher the concentration, the darker the color. This is because a thermal equilibrium exists between the colorless spirooxazine and the colored ring-opened form. At a fixed concentration, the absorption intensity of the colored spirooxazine will increase as the temperature rises. The color of ring-opened spirooxazine compounds will deepen in the melting state, with colors that are usually purple or blue.

### 2.5. Comparison between Spiropyrans and Spirooxazines

The comparisons between spiropyrans and spirooxazines focus on light fatigue resistance and the influence of the environment.

#### 2.5.1. Light Fatigue Resistance

For spirooxazines, thousands of cycle times between the ring-opened, colored forms and ring-closed, colorless forms can be obtained by photoinduction. No new products or intermediate substances are generated when light irradiation is stopped. However, the fatigue of spiropyrans is generally not very good. Usually, the cycles between the colorless and the colored spiropyrans occur approximately 100–1000 times, and only a few can reach more than 10,000 cycles [49]. Raymo studied many isomerization processes of fluorescent systems incorporating both a spiropyran and a fluorescent Bodipy. From the experimental data, they found that the amount of energy which decreased cannot be restored completely after light irradiation cycles. The more times irradiation occurs, the more the energy decays; this indicates that the structural transformations of spiropyrans are accompanied by energy degradation [50,51,52]. To address the above problem, Saad et al. designed and successfully prepared a covalent spiropyran-polyoxometalate-Bodipy tricomponent hybrid. A polyoxometalate core can prevent the gradual photodegradation of fluorescence for the spiropyran and Bodipy be consistent system. Compared with other spiro compounds and Bodipy systems, such combinations of spiropyran, polyoxometalate and Bodipy possess a high photofatigue resistance [53]. The reason for light fatigue is the co-occuring side reactions. Because the intermediate of the ring-opened reaction is mostly based on a zwitterion structure and the charge is concentrated, it is vulnerable to environmental factors that lead to energy consumption [54,55]. For example, oxygen in the air encourages photooxidation, which is the result of the reaction between the triplet state of the ring-closed SP form and oxygen [56,57].

#### 2.5.2. Influence of the Environment

Here we focus on discussing the changes of two environmental parameters: temperature and polarity. As we all know, heating will produce a certain thermal bleaching effect on the chromophores, which limits the maximum color intensity obtained at a given temperature [58]. Spirooxazines can be stored for a long time at room temperature, which does not influence the photochromism properties even under wet conditions and higher temperature (>50 °C). However, it is reported that spiropyrans exhibit poor stability when the environmental temperature changes.

When the solvent polarity changes, spirooxazines are not much affected; however, the photochromism properties of spiropyran compounds are reduced or lost when dissolved in polar solvents such as ethanol and acetone. Moreover, spirooxazines show good photochromism when they are mixed with a polymer medium but spiropyrans do not. Therefore, many applications of spiropyran compounds are restricted due to their poorer chemical stability. Spirooxazines have been used more in rewritable optical storage materials, optical control switches, variable optical density filter elements, and so on [9,59].

## 3. Applications Based on Composite Structures of Spiropyrans and Spirooxazines with Fluorescent Material

Due to their excellent photochromism properties, various approaches have been devised to create multifunctional materials based on spiropyrans and spirooxazines. The most investigated principle is the fluorescence resonance energy transfer (FRET) between the spiropyrans and spirooxazines MC forms and fluorescent molecules, because the ring-opened MC form has an intense absorption in the visible light region [60,61,62]. Efficient FRET happens when the emission spectra of fluorescent molecules overlap with the absorption spectra of the MC forms of spiropyrans and spirooxazines under the conditions of controlled distance and molecular orientation. Additionally, spiropyrans and spirooxazines could also respond to other stimuli such as pH, temperature, stress, and so on, as well exhibit distinctive absorption characteristics after treatment with different external stumuli. Energy transfer based on the above processes are employed in various composite structures. Introduction of spiropyrans and spirooxazines to construct energy transfer systems could not only modulate the fluorescence of the whole system but may also be used in different kinds of chemical sensing, fluorescence probes, optical modulators and switches, and so on.

### 3.1. Chemical Sensing

**Figure 3:**
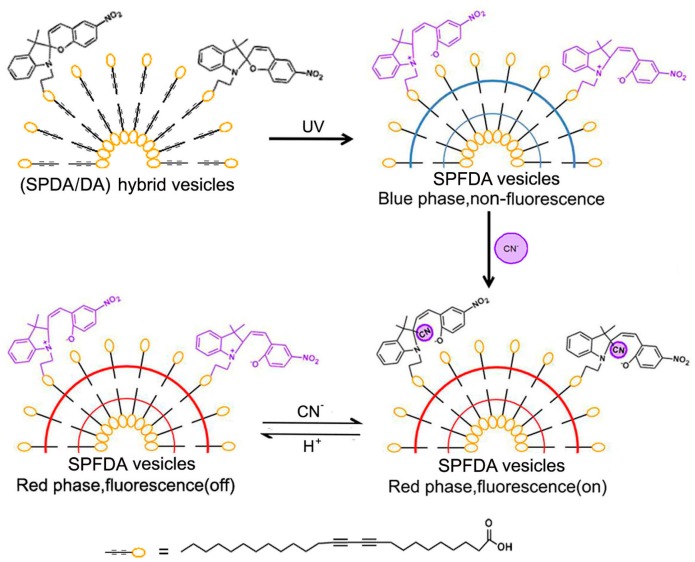
Schematic representation of highly selective, sensitive, and reusable detection of CN^−^ by the composite vesicles based on spiropyran in aqueous solution (reprinted with permission from Xia et al. [63]).

Our group fabricated spiropyran-functionalized polydiacetylene (SFPDA) vesicles which are used for the selective and sensitive detection of cyanide anions (CN^−^) [63]. We synthesized spiropyran-substituted diacetylene through the esterification reaction between hydroxy and carboxyl groups. Then, the monomers diacetylene and spiropyran-substituted diacetylene were prepared in a compound vesicle (SFPDA) where the spiropyran can maintain its multi-stimuli responsive properties. As shown in Figure 3, the colorless transparent vesicle solution changed to blue under UV light radiation. From the absorption spectra, it is evident that the monomer diacetylene polymerized into polydiacetylene (PDA) and that the spiropyrans in the shell of the vesicle were converted to the opened MC form.

Under visible light irradiation, MC could reversibly transform to SP and not affect the properties of PDA. After the addition of CN^−^, the blue vesicle solution changed to red gradually and the intensity of absorption peak at 640 nm dramatically decreased. Meanwhile, new absorption peaks appeared at 540 and 500 nm of the absorption spectra. The mechanism for the selective detection of CN^−^ using SFPDA vesicles was the nucleophilic addition reaction between CN^−^ and MC, which induces the strains and distortions of PDA chains and leads to the color and spectral changes of the whole solution. Additionally, as we know, the “blue-phase” PDA is non-fluorescent; however, their “red-phase” counterparts fluoresce. The fluorescence changes of the entire system could also be used to indicate the detection process along with the color changes. After adding CN^−^, fluorescence appeared due to the “blue-phase” PDA turning to “red-phase”. Fluorescence was quenched upon treatment with H^+^ because the interaction between H^+^ and CN^−^ was stronger than between MC and CN^−^. This removed the CN^−^ from the designed probe, leading to the FRET between MC and the “red-phase” PDA; thus, the fluorescence of the whole system decreased.

Experimental results showed that CN^−^ was detected by the composite vesicle solution based on spiropyran; an obvious color change and detection time were obtained within one minute. Notably, the limit of detection concentration was found to be very low (about 0.5 µM). At the same time, the whole detection process was not disturbed by other anions of the same kind. Also, the nucleophilic addition reaction of spiropyran with CN^−^ can be easily blocked through simple acid treatment. In conclusion, the designed probe allowed a highly selective, sensitive, and reusable detection of CN^−^ in water using colorimetric and fluorimetric changes.

The MC form of spiropyrans and spirooxazines also always emits fluorescence under excitation with proper light. Thus, without combining them with other fluorescent units, spiropyrans and spirooxazines are also used in chemical sensing assays based on the change of their own fluorescence. For example, Huang reported that when different metal ions were added to a spirooxazine ethanol solution, the responses of the absorption and fluorescence spectra were different [64]. Under dark, UV, and visible light irradiation, complexes of spirooxazine and various metal ions displayed various fluorescent signals, such as fluorescence enhancement or a shift of fluorescence spectra. Furthermore, they designed a microchip using spirooxazine as the sensor, allowing for multiple metal ion detection through analyzing the fluorescence signals. In order to show the differences intuitively, the authors used linear discriminant analysis (LDA) to evaluate the response data.

**Figure 4:**
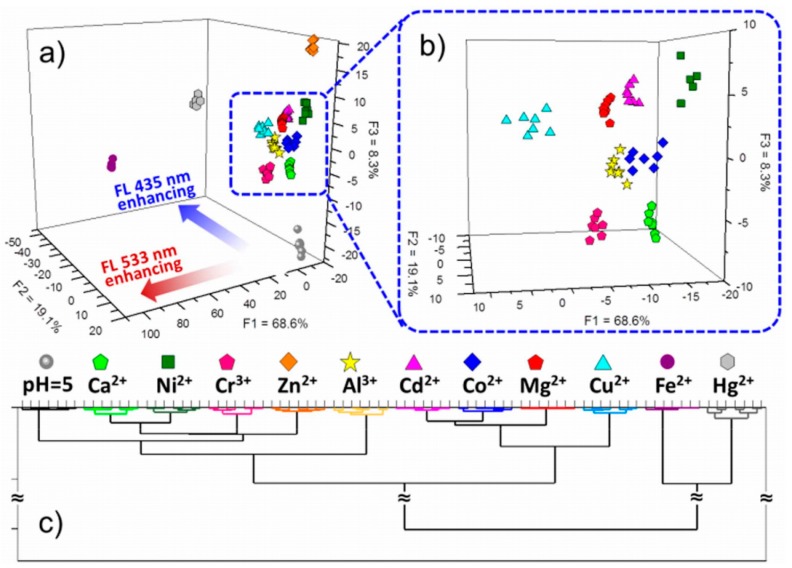
Fluorescent discrimination of 11 metal ions based on spirooxazine microchip. (**a**,**b**) Graphs of linear discriminant analysis (LDA) result, showing a clear clustering of the 11 metal ions and its corresponding magnified images. LDA reflected the metal ions’ specific fluorescent enhancement at 435 nm or 533 nm; (**c**) Heirarchal clustering analysis (HCA) gave the similarity clustering of the analyzed ions based on the fluorescence variation trend of the spirooxazine-metallic coordination (reprinted with permission from Huang et al. [64]).

As shown in Figure 4, the distance of the clusters in the LDA score plot represented differential fluorescent signals after combination with different metal ions. We can see that one control sample (water pH = 5.5) and eleven metal ion samples (Al^3+^, Cd^2+^, Co^2+^, Cr^3+^, Cu^2+^, Fe^2+^, Hg^2+^, Mg^2+^, Zn^2+^, Ni^2+^ and Ca^2+^) were obviously separated in the space distribution. Also, they used hierarchical clustering analysis (HCA), as shown in Figure 4c, for in-depth analysis. This method can discriminate hundreds of similar analytes, expanding the application scopes greatly. Through the combinations of LDA and HCA, various metal ions have been subtly and accurately detected.

### 3.2. Fluorescence Probes

**Figure 5:**
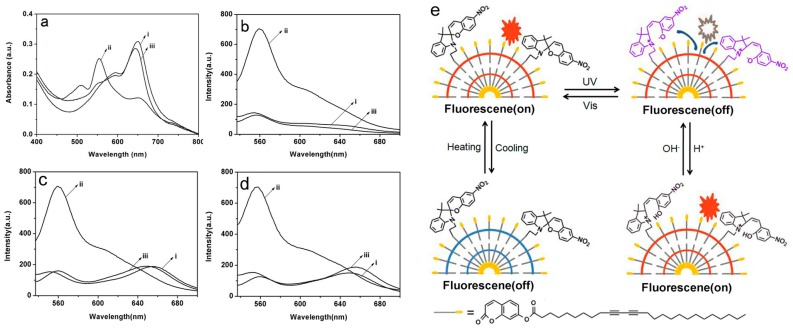
(**a**) UV–visible absorption spectra and (**b**) fluorescence spectra of the hybrid vesicles solution: (i) before treatment, (ii) upon heating, and (iii) cooled back to room temperature; (**c**) Fluorescence spectra of the hybrid vesicles solution at 70 °C, then under 365 nm irradiation: (i) before treatment, (ii) upon 435 nm light irradiation, and (iii) upon 365 nm light irradiation again; (**d**) Fluorescence spectra of the hybrid vesicles solution at 70 °C, then under 365 nm irradiation: (i) before treatment, (ii) upon addition of H^+^, and (iii) upon addition of OH^−^; (**e**) Scheme of the reversible multi-stimuli-responsive spiropyran-based fluorescence probe (reprinted with permission from Xia et al. [65]).

Similar to Section 3.1, we synthesized fluorescent probes through incorporating spiropyran groups into coumarin-substituted polydiacetylene (CO-PDA) vesicles which were multi-stimuli-responsive, rapid, and low-cost [65]. Reversible transformations of the colored and colorless form of spiropyran were used as the driving force to control the FRET between the MC form of spiropyran and the “red-phase” PDA. This is because the absorption spectra of the colored MC overlap with the “red-phase” PDA fluorescence spectra (Figure 5). Specifically, like the preparation of composite vesicles in Section 3.1, the monomers spiropyran-substituted diacetylene (SPDA) and coumarin-substituted diacetylene (CODA) were fabricated into multifunctional hybrid vesicles. After undergoing UV light irradiation at room temperature, spiropyrans transferred from SP to MC, and DA polymerized into polymer PDA. Upon treatment with visible light, the main chains of PDA remained unchanged while the absorption spectra of MC decreased. When treated with UV light again, the intensity of absorption spectra for MC returned to the original value. Also, upon treating with H^+^, the colored MC form isomerized to the MCH form due to the protonation reaction; the reversible structural transformation happened upon addition of OH^−^.

As mentioned above, PDA is fluorescent in the “red-phase” after the “blue-phase” is heated. The fluorescence can be quenched after UV light irradiation due to the FRET between the MC form of spiropyran and the “red-phase” PDA. Then, upon treating with H^+^, the intensity of fluorescence returned back; this was because no FRET exists between the “red-phase” PDA and the protonated MCH form of spiropyran, which has no absorption in the 500 nm to 600 nm region. Combined with the reversible thermochromism resulting from the strong head group interactions of CO-PDA, the prepared hybrid fluorescence probe could response to light, thermal, and pH stimuli simultaneously. Moreover, the above processes were completely reversible. The prepared reversible colorimetric and fluorescence probe can be used in sensing for complicated micro-environments of biological systems and in the construction of various molecule memory elements for information storage, optical recording, and so on.

### 3.3. Optical Modulators

**Figure 6:**
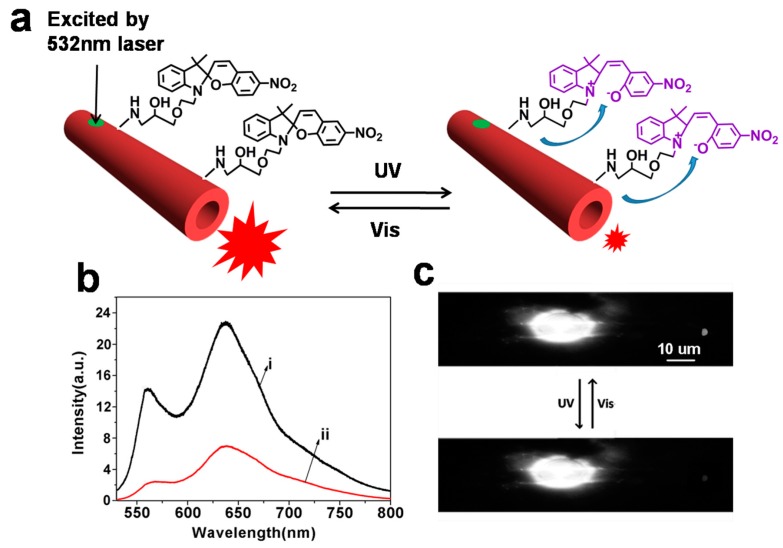
(**a**) Modulation of waveguiding in spiropyran-functionalized microtubes; (**b**) Tip emission spectra of the SFPDA microtubes (i) before and (ii) after UV light irradiation; (**c**) Fluorescence microscope images collected upon excitation of the same SFPDA microtube following visible and UV light irradiation (reprinted with permission from Xia et al. [66]).

Based on the FRET between spiropyrans and fluorescent molecules, we observed the optical modulation of waveguiding in one-dimensional conjugated polymer microtubes [66]. Spiropyran-functionalized PDA (SFPDA) microtubes were fabricated by incubating self-assembled microtubes in an epoxy-substituted spiropyran solution. Upon treatment with UV light, the surface spiropyran units isomerized from SP to MC, the absorption spectra of which overlaps with the fluorescence of the “red-phase” PDA. The fluorescence intensity of the microtubes decreased dramatically, resulting in a reduction of the tip waveguiding. Under visible light irradiation, MC transformed into SP and the fluorescence intensity and tip waveguiding for the SFPDA microtubes returned to the original value. This is because there was no FRET between SP and the “red-phase” PDA (Figure 6a,b). Thus, the reversible transformations of spiropyran between the SP and MC form can be used to modulate the waveguiding properties of SFPDA microtubes. Similar fluorescence changes of the tip can be observed after further regeneration and modulation cycles, as shown in Figure 6c.

Also, we constructed optical logic operations in the above functionalized microtubes based on the reversible structural isomerizations between the colorless SP, protonated MCH, and purple MC form of spiropyran under light, thermal, or pH stimuli. The mechanism was the same as described in Section 3.2. Combinations of different stimuli were utilized as the input signals, and the tip emission of SFPDA microtubes was taken as the output signal. Modulations of the fluorescence and tip emission of the SFPDA microtubes were realized through controlling the FRET between the MC form and PDA matrix, which was induced by isomerizations of spiropyran. The controllable and resettable logic gate operations of SFPDA microtubes paved the way for future miniaturized optoelectronic elements for photonic circuits, ultrafast information processing, optical computing, and sensing.

### 3.4. Optical Switches

**Figure 7:**
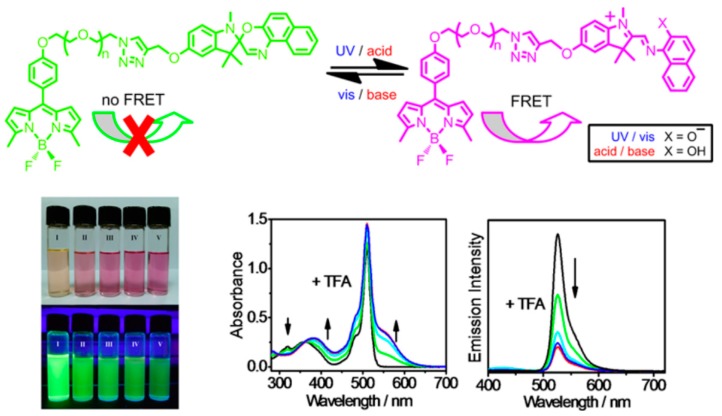
Scheme of the optical switch based on the spirooxazine-containing Bodipy derivatives. (reprinted with permission from Kong et al. [67]).

Spirooxazines exhibit distinctive absorption characteristics after changing environmental parameters such as light, pH, and temperature. These properties were employed to investigate FRET in composite particles, nanotubes, and many other constructed structures, as well asused in the form of various optical switches. Kong et al. combined the fluorescent molecule Bodipy with spirooxazines and synthesized two series of spirooxazine-containing Bodipy derivatives [67]. They studied the on and off switching of fluorescence for different structures through FRET between Bodipy and spirooxazines, which could pave the way for the future design of novel multifunctional optical switches. As shown in Figure 7, the whole system exhibited bright green fluorescence under photoexcitation due to Bodipy. However, after UV light irradiation or treatment with acid, spirooxazines transformed from the SP form to MC, and FRET occurred between MC and Bodipy. This led to the quenching of green fluorescence and the generation of red fluorescence, which was ascribed to the MC form of spirooxazine. Due to the reversible structural transformations, they obtained the green fluorescence again after the solution was treated with visible light or base. The above process can be repeated many times without energy degradation, so the isomerization of spirooxazine could be used to switch the fluorescence of the whole system. The designed Bodipy-containing spiropyran and spirooxazine derivative materials are very versatile and could be utilized in a variety of different fields, such as multifunctional switches, solar energy conversion sensitizers, cation fluorescence labels, and sensors.

## 4. Conclusions and Outlook

This review has discussed recent progress in spiropyrans and spirooxazines. Their structural features and reversible isomerization properties are introduced in detail. Various applications based on FRET of the ring-opened forms of spiropyrans and spirooxazines with fluorescent molecules have been described.

After analyzing the application prospects, we put forward some new viewpoints. We look forward to a substantial improvement in the performances of all aspects of spiropyrans and spirooxazines in the future, such as improved light fatigue in order for better application in data storage and optical switches. Also, the properties of substituents have a great influence on the overall performance, so further exploring electron-donating or electron-withdrawing substituents will help to greatly improve performance. Furthermore, fixed timing control for photoisomerization is of greater importance as it allows control of modulation for various processes based on spiropyrans and spirooxazines. Another direction will be structural modification in order to use spiropyrans and spirooxazines in different kinds of media, especially the aqueous media where it will be most needed in biological systems. Additionally, the design and development of more composite systems of spiropyrans and spirooxazines with fluorescent molecules would be useful in constructing different kinds of fluorescent sensors, advanced optical components, and devices. This aspect is very challenging and worthy of further study.
